# Phenotypic and molecular characterisation of *Brucella *isolates from marine mammals

**DOI:** 10.1186/1471-2180-8-224

**Published:** 2008-12-17

**Authors:** Claire E Dawson, Emma J Stubberfield, Lorraine L Perrett, Amanda C King, Adrian M Whatmore, John B Bashiruddin, Judy A Stack, Alastair P MacMillan

**Affiliations:** 1 Department of Statutory and Exotic Bacterial Diseases, Veterinary Laboratories Agency, New Haw, Addlestone, Surrey, KT15 3NB, UK; 2 FMD Pathogenesis Group, Institute for Animal Health, Pirbright Laboratory, Ash Road, Pirbright, Woking, Surrey, GU24 ONF, UK

## Abstract

**Background:**

Bacteria of the genus *Brucella *are the causative organisms of brucellosis in animals and man. Previous characterisation of *Brucella *strains originating from marine mammals showed them to be distinct from the terrestrial species and likely to comprise one or more new taxa. Recently two new species comprising *Brucella *isolates from marine mammals, *B. pinnipedialis *and *B. ceti*, were validly published. Here we report on an extensive study of the molecular and phenotypic characteristics of marine mammal *Brucella *isolates and on how these characteristics relate to the newly described species.

**Results:**

In this study, 102 isolates of *Brucella *originating from eleven species of marine mammals were characterised. Results obtained by analysis using the Infrequent Restriction Site (IRS)-Derivative PCR, PCR-RFLP of outer membrane protein genes (*omp*) and IS*711 *fingerprint profiles showed good consistency with isolates originating from cetaceans, corresponding to *B. ceti*, falling into two clusters. These correspond to isolates with either dolphins or porpoises as their preferred host. Isolates originating predominantly from seals, and corresponding to *B. pinnipedialis*, cluster separately on the basis of IS*711 *fingerprinting and other molecular approaches and can be further subdivided, with isolates from hooded seals comprising a distinct group. There was little correlation between phenotypic characteristics used in classical *Brucella *biotyping and these groups.

**Conclusion:**

Molecular approaches are clearly valuable in the division of marine mammal *Brucella *into subtypes that correlate with apparent ecological divisions, whereas conventional bioyping is of less value. The data presented here confirm that there are significant subtypes within the newly described marine mammal *Brucella *species and add to a body of evidence that could lead to the recognition of additional species or sub-species within this group.

## Background

The isolation of *Brucella *from marine mammals and the classical biotyping of these strains were first reported in 1994 coincidently in two locations. Ross *et al*. (1994) [1] reported the isolation of *Brucella *from tissues collected *post mortem *from stranded marine mammals including common seal (*Phoca vitulina*), harbour porpoise (*Phocoena phocoena*) and common dolphin (*Delphinus delphis*) around the coast of Scotland whilst Ewalt *et al*. (1994) [2] reported the recovery of the organism from an aborted foetus of a bottlenose dolphin (*Tursiops truncatus*) in the USA. The isolation of *Brucella *has since been described from a wide variety of marine mammals including Atlantic white-sided dolphin (*Lagenorhynchus actus*), striped dolphin (*Stenella coeruleoalba*), hooded seal (*Crystophora cristata*), grey seal (*Halichoerus grypus*), European otter (*Lutra lutra*) [3,4], Pacific harbour seal (*Phoca vitulina richardsii*) [5], minke whale (*Balaenoptera acutorostrata*) [6] and white beaked dolphin (*Lagenorhynchus albirostris*) [7].

Although the number of isolations of *Brucella *strains from marine mammals are still relatively limited, there is strong serological evidence that such infections are widespread, in prevalence, in the variety of species infected and in their geographical distribution. Evidence arising from the northern hemisphere is particularly well documented from locations such as the Scottish coast [8], the coasts of England and Wales [9], the north Atlantic Ocean, west of Iceland to the north of Norway and Russia [10], the Mediterranean Sea [11], Arctic Canada and the Atlantic coast of North America [12,13]. Evidence from the southern hemisphere is less well documented although there are reports of positive serological results from baleen whales (*Mysticeti*) from the western North Pacific [14], Hawaiian monk seals (*Monachus schauinslandi*) from the Northwestern Hawaiian Islands [15], bottlenose dolphins from the Solomon Islands [16], cetaceans living off the Peruvian coast [11] and seals from Antarctica [17] and Australia [18].

For many years, the accepted taxonomy of *Brucella *comprised six species: *B. abortus, B. melitensis, B. suis, B. ovis, B. canis *and *B. neotomae*, some of which are further divided into biovars. The attribution to species is usually made using classical biotyping techniques to identify phenotypic characteristics such as CO_2 _dependency, substrate utilisation, dye and antibiotic susceptibility, phage lysis, and serotyping [19,20]. Such phenotypic species classification relates closely to host preference and therefore this system of classification has assisted in the study of *Brucella *and remains widely used today for epidemiological purposes. DNA-DNA hybridization studies have demonstrated that *Brucella *is a highly homogeneous genus (>90% DNA-DNA relatedness) [21-25]. Nevertheless, the development of a range of DNA based typing techniques [26-34] have supplemented the classical techniques and show remarkable correlation with the classical *Brucella *species.

Much attention has been focussed on attempts to classify the marine *Brucella *strains in a way consistent with the framework of the existing six species. Studies using classical biotyping methods revealed characteristics which were typical of the genus *Brucella *[19] but the pattern of which differed from those displayed by the currently recognised species [2,4]. In particular, the oxidative metabolism tests have allowed the differentiation of a number of distinct marine specific phenotypes [4]. Such studies resulted in a number of tentative proposals to define the *Brucella *isolated from marine mammals as separate species [4,20,35,36] and much additional investigation has been completed in an attempt to differentiate the strains and gain insight to their evolutionary relationship with the existing recognised species. A number of early studies were carried out using classical biotyping, PCR-RFLP of the *omp*2a gene [37], Pulsed Field Gel Electrophoresis (PFGE) [38] and IS*711 *fingerprinting [6,35].

Recently, following analysis of a small number of strains, two new species names, *Brucella ceti *and *Brucella pinnipedialis *were validly published for isolates from cetaceans and pinnipeds respectively [36]. Nevertheless, the current status of the classification of *Brucella *isolated from marine mammals remains controversial with molecular evidence suggesting that *B ceti *comprises two genetic clusters [32,39,40]. This study aims to characterise *Brucella *isolates from a range of marine mammals originating from various geographical locations using a selection of widely recognised classical and molecular techniques in direct comparison. The data generated may assist in resolving some of these taxonomic issues and gives the most extensive description to date of the characteristics of this group of isolates.

## Results

A comparative summary of the results of characterisation of all 102 isolates by both phenotypic and molecular approaches is provided in Table 1 (Additional file 1.)

### Molecular characterisation

#### PCR-RFLP of *omp *genes

Analysis of the 102 isolates examined in this study generated six unique profiles. Of the six profiles M(J) was the most common being present in 53/102 isolates. This profile was predominantly associated with porpoises (79% of isolates) but was also less frequently seen in dolphins (15%), seals (4%) and whales (2%). Of the remaining five profiles, N(K) was exclusively associated with dolphins while profiles L(I) and O(I) were predominantly associated with seals (95% and 83% of isolates respectively). Two less commonly observed profiles, P(I) and Q(I), were seen only in hooded seal isolates and in a single isolate from a bottlenose dolphin respectively.

### IS*711 *fingerprinting

**Figure 1:**
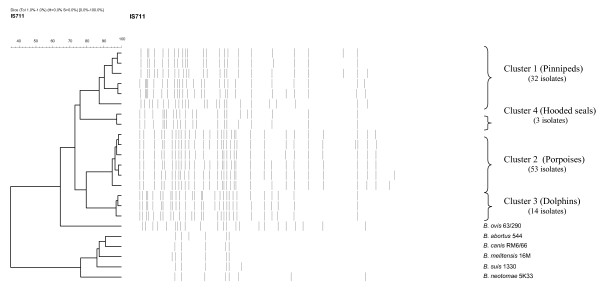
Representation of examples of full IS*711 *fingerprints of *Brucella *isolates from marine mammal and reference strains.

IS*711 *fingerprinting analysis of the 102 isolates generated 17 unique marine-specific IS*711 *profiles of which 13 were observed amongst the European strains and four amongst the USA strains. All fingerprints comprised more than 20 bands and examples of each profile are shown in Figure 1. The distribution of IS*711 *copies in the genomes of all of the marine strains included in this study showed little resemblance to any pattern previously observed in any other species or biovars, including those of *B*. *ovis *or *B*. *suis *bv 2 which themselves have many copies of the element. Clustering of the 17 fingerprints divided them into 4 groups (labelled clusters 1 – 4) when using a cut-off value of >85% similarity. Members of cluster 1 were isolates predominantly associated with seals possessing *omp *pattern L(I) or O(I). Members of cluster 2 were isolates predominantly associated with porpoises possessing *omp *pattern M(J). Members of cluster 3 were isolates of *omp *pattern N(K) exclusively associated with dolphins. Cluster 4 isolates, representing *omp *pattern P(I), were associated only with hooded seals.

#### IRS-Derivative PCR

The ISR-Derivative PCR examines the presence/absence of four distinct PCR fragments in isolates. Positive results for PCRs II and III were always congruent with each other and with IS*711 *fingerprinting cluster 2, comprising of predominantly porpoise isolates. PCR IV was positive only in the group exclusively associated with dolphins corresponding to IS*711 *fingerprinting cluster 3. PCR I appeared to be specific for strains predominantly originating from seals (IS*711 *fingerprinting clusters 1 and 4). Four isolates repeatedly failed to provide a product with any of the PCRs. These were 3 isolates from IS*711 *fingerprinting cluster 1 all originating from the USA and a single hooded seal isolate from the UK.

#### Phenotypic characterisation

Classical bacteriological biotyping techniques to identify phenotypic characteristics have traditionally been used to classify *Brucella *strains. Although these techniques have proved useful, they are subjective and it is well recognised that some strains may give unexpected or anomalous results to some or all of the tests.

#### CO_2 _requirement

In this study, isolates originating from seals (corresponding to IS*711 *fingerprinting cluster 1) in general showed a requirement for CO_2 _for growth whereas isolates from cetaceans (IS*711 *fingerprinting clusters 2 and 3) generally did not. However several exceptions were observed (Table 1, Additional file 1).

#### Agglutination with A and M monospecific antisera

Serotyping with A and M monospecific antisera is very subjective and it is well recognised that apparently anomalous results are observed. In addition, strains converting to the rough phenotype on subculture may lose the characteristic. The majority of isolates were A dominant only but some strains agglutinated both A and M antisera. These strains did not correspond to a single molecular type, but were distributed throughout the molecular groups discussed above. None of the isolates originating from the USA agglutinated with either the A or M monospecific serum (Table 1, Additional file 1).

#### Phage lysis

Smooth *Brucella *strains are lysed by specific phages, and the pattern of lysis is an important characteristic which has been used to classify terrestrial strains, although the characteristic may be lost if the strains become rough on subculture. Phage lysis results among the panel examined here were very variable with no clear patterns correlating to the groups identified by the various molecular typing methods. The majority of isolates (from all IS*711 *fingerprinting clusters) were lysed by phage Bk_2 _and of the isolates that showed any lytic activity only three isolates (29, 34 and 102) from IS*711 *fingerprinting clusters 1 and 2 were not lysed by this phage. Sixty six (65%) of isolates were lysed by Wb phage, forty-eight (47%) were lysed by Fi phage and eleven (11%) of isolates were lysed by Tb phage. A total of six isolates all originating from IS*711 *fingerprinting cluster 2 showed no lysis by any of the phages.

#### Dye sensitivity

The vast majority of isolates were not inhibited by basic fuchsin or thionin. A few isolates of IS*711 *fingerprinting cluster 2 were inhibited by both fuchsin and thionin (7, 10, 15) or only by fuchsin (9, 20).

## Discussion

Data from a large comparative analysis of molecular and phenotypic characteristics of marine mammal *Brucella *is presented here. The 102 strains included in this study are derived from the North Atlantic from locations around the coasts of the UK, France, Spain, Germany, and Norway. Although they are predominantly European in origin a small number of North American isolates are also included in this study and two from the Pacific ocean. Wherever serological surveys have been carried out, a high prevalence has been found in Pacific waters [11,17,18]. In addition, a strain isolated from a human brucellosis case in New Zealand gave test results indistinguishable from one of the marine mammal strains (97) included in this study originating from a bottlenose dolphin from the USA [41]. Recent analysis of two further historical cases of severe human brucellosis originating in Peru [42] also revealed that both associated isolates share the same genotype [43]. These findings indicate that marine mammal *Brucella *isolates may also be common in parts of the world where little data has yet been reported. Until recently molecular evidence suggested that *Brucella *originating from marine mammals from the Pacific ocean all belonged to *B*. *pinnipedialis *[41,44]. However, a recent report provides evidence that strains belonging to *B*. *ceti *are present [45]. This report is also corroborated by our own studies (unpublished data).

**Figure 2:**
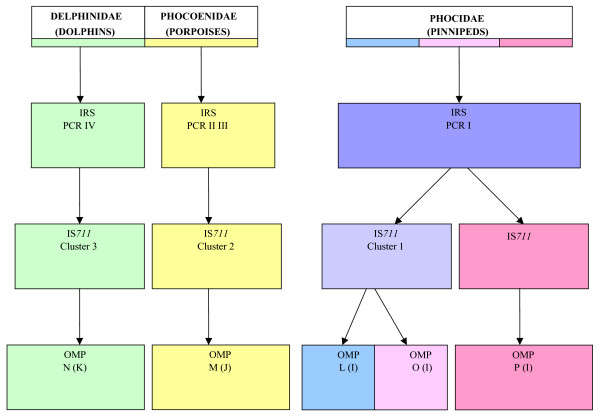
**Summary of relationships between *Brucella *isolates based on molecular tests**. This schematic is based on observations of strains isolated from European waters as too few isolates from elsewhere have been received to date to reach reliable conclusions.

When considered in full, the data obtained in this study reveals a remarkable congruence between groups identified by different molecular tests and with the order and/or species of marine mammal from which they originated. These relationships are depicted graphically in Figure 2. Thus, when considering IS*711 *fingerprints, four main clusters were identified. Within the numerically largest of these clusters, cluster 2, all isolates possess *omp *PCR-RFLP pattern M(J), and display the IRS-PCR profile - + + -. The vast majority of these strains originate from harbour porpoises. In contrast members of IS*711 *fingerprinting cluster 3 all possess *omp *PCR-RFLP profile N(K) and IRS-PCR profile - - - +. Without exception these isolates originate from dolphins. Thus cetacean isolates appear to fall into two well-separated clusters with different preferred hosts that are consistently apparent using different molecular approaches. These isolates correspond to the newly described species *B. ceti*.

Isolates that are members of IS*711 *fingerprinting cluster 1 correspond to two main *omp *PCR-RFLP profiles L(I) and O(I). The vast majority of these isolates possess IRS-PCR profile + - - -, although three isolates within this group, originating from the Atlantic coast of the USA, reacted with none of the IRS-PCR primer sets (profile - - - -). A single isolate also possesses a unique *omp *PCR-RFLP profile Q(I). Members of this IS*711 *fingerprinting cluster were predominantly isolated from seals and correspond to the recently described *B. pinnipedialis*. Three isolates obtained from hooded seals comprised IS*711 *fingerprinting cluster 4. These three isolates also possess a unique *omp *PCR-RFLP profile P(I) and, as with members of IS*711 *fingerprinting cluster 1 possess IRS-PCR profiles + - - - or - - - -.

Thus, the overall finding of four groups identified using a cut-off value of >85% similarity to define IS*711 *fingerprinting clusters is consistent with other molecular tests and with host preferences observed in this study. Furthermore, the grouping of strains in this study is consistent with other recent studies. Studies based on genome mapping recently divided marine mammal *Brucella *strains into three major groups [40]. Strains originating from seals fell into one group (Group I), with a single hooded seal isolate placed within this group, although possessing a variant profile. As in this study isolates from cetaceans fell into two distinct groups comprising isolates from common and striped dolphins (Group II) and isolates predominantly from harbour porpoises (Group III). The authors of this article suggested that, given the large branch lengths between the three groups, they could be classified as separate species. Moreover, a recent comparative study using Variable-Number-of Tandem-Repeats (VNTR) analysis and multilocus sequence analysis (MLSA) divides strains into the same three groups [39]. This study identified congruent VNTR and MLSA groups corresponding to isolates with preferred hosts of dolphins, porpoises and seals. Furthermore, within the seal group isolates from hooded seals formed a subgroup, as seen in this study, and isolate 97, corresponding to the isolate with a distinct *omp *PCR-RLP profile, was found to represent a unique sequence type and form a separate sub-branch within the group based on VNTR clustering. While agreeing with the validity of a species group comprising of seal isolates and now classified as *B. pinnipedialis*, the authors of this manuscript questioned the validity of the group since published as *B. ceti*. This was based on the clear division into two groups with distinct host specificities that appeared less closely related to each other than either was to the group of seal isolates now classified as *B. pinnipedialis*.

While there was clear division into consistent groups based on molecular typing in combination with apparent host preferences there was little evidence of any consistent phenotypic traits based on the conventional biotyping procedures usually applied to *Brucella*. Although classical bacteriological typing has been universally used for identifying and characterising *Brucella *strains isolated from terrestrial mammals, the results obtained in this study were disappointing. It is well recognised that such methods require considerable skill and expertise and correctly standardised reagents to carry out, that their interpretation is somewhat subjective, and that anomalous results are not uncommon. However, the results of phage lysis, sensitivity to dyes and agglutination with monospecific sera showed no evidence of any grouping patterns that corresponded to host of origin or the molecular groups described above. The results observed in this study suggest that these tests offer little value in the characterisation of marine mammal strains. The strongest correlation was with the requirement of CO_2 _for growth. In general, isolates derived from seals required additional CO_2 _for growth, but isolates from cetaceans did not, although there were exceptions. This is in agreement with the observations of Foster *et al*. (2007) [36] in the description of *B. ceti *and *B. pinnipedialis*. However, a number of other characteristics outlined in the species descriptions are not entirely consistent with the data presented here. Thus, both *B. ceti *and *B. pinnipedialis *are described as A antigen dominant but isolates included in this study, corresponding to both of these species, react with monospecific sera to both A and M antigens. Furthermore isolates of *B. ceti *are described as being lysed by Wb phage but not lysed by Tb phage. However, in this study we found exceptions to this description for both Wb phage (in IS*711 *fingerprinting cluster 2 isolates) and Tb phage (in both IS*711 *fingerprinting clusters 2 and 3) with reproducible results. Similarly isolates of *B. pinnipedialis *are described as being lysed by Wb phage with 'a small number' of isolates being lysed by Tb phage. While results reported here agree with the latter observation there are many examples in this study of isolates corresponding to *B. pinnipedialis *not being lysed by Wb phage (IS*711 *fingerprinting cluster 1).

## Conclusion

This study represents the most extensive characterisation of marine mammal strains, based on traditional *Brucella *bioyping approaches and molecular approaches, carried out to date. The marine mammal *Brucella *strains described in this paper could readily be distinguished from terrestrial *Brucella *species using *omp *PCR-RFLP, IS*711 *fingerprinting, and IRS-derivative PCR. However, characteristic profiles based on conventional biotyping were not apparent. Using molecular methods, strains fell into rational groups, with good congruence between methods and with the preferred host. Strains originating from cetaceans clearly fall into two groups with either dolphins or porpoises as their preferred host. These findings are consistent with previous suggestions, based on other molecular evidence [39,40], that the recently described species *B. ceti *could be further subdivided. Strains from seals fell into one major group corresponding to the recently described species *B. pinnipedialis*. However, a small number of strains examined from hooded seals, while clustering most closely to *B. pinnipedialis*, were quite distinct from the other seal strains using *omp *PCR-RFLP and IS*711 *fingerprinting. Again this is consistent with observations elsewhere [39,40] and suggests that there are also further ecological subdivisions within pinniped *Brucella *isolates. While a large number of isolates were characterised in this study, their geographical distribution is limited, and it is clear that much more extensive and global surveillance is required in order to fully understand the distribution, ecology and genetic relatedness of *Brucella *isolates from marine mammals.

## Methods

### *Brucella *isolates

A total of 102 *Brucella *strains isolated from marine mammals were included in this study. Cetacean isolates included organisms obtained from 42 harbour porpoises, seven Atlantic white-sided dolphins, one white-beaked dolphin, three bottlenose dolphins, four common dolphins, eight striped dolphins, two minke whales, and one unspecified dolphin species. Pinniped isolates included organisms originating from 22 common seals, three grey seals, three hooded seals and five unspecified seals. A single isolate originating from a European otter was also included. Reference and type strains of currently recognised species of *Brucella *used in this study were all obtained from the VLA culture collection. All isolates had previously been lyophilized and stored at 2–8°C. The isolates were subcultured onto serum dextrose agar (SDA) plus 10% equine serum and incubated at 37°C for 3 to 5 days in the presence of additional 10% CO_2_.

### Classical biotyping

**Phenotypic **characterisation of the isolates was carried out using internationally recognised classical biotyping methods including the requirement for additional CO_2 _for growth, sensitivity to aniline dyes, serotyping using A and M monospecific sera and phage typing [19,20].

### Preparation of genomic DNA

Genomic DNA was prepared from 3–4 day old cultures on SDA as described previously [29].

### PCR-RFLP of *omp*2a, *omp*2b and *omp*25 genes

PCR-RFLP of the genes encoding outer membrane proteins Omp2a, Omp2b and Omp25 was conducted essentially according to the method described by Cloeckaert *et al*., (1995) [28]. *Brucella *reference strains were included to maintain consistency of pattern nomenclature (data not shown). PCR products were digested with enzymes previously shown to discriminate within the marine mammal *Brucella*. Thus *omp*25 was digested with *Eco*RV, *omp*2a was digested with *Hinf*I and *Kpn*I, while *omp*2b PCR products were digested with *Bgl*II, *Taq*I, *Hae*III, *Hinf*I, *Kpn*I, and *Eco*RI. PCR products of isolates that possessed the O(I) profile were further treated with the enzyme *Alu*I in order to differentiate them from the P(I) group. RFLP profiles of the closely related *omp*2a and 2b genes of each *Brucella *reference strain have been analysed and described previously [28,46]. These papers used the letters A through to K to represent each profile with each letter representing a certain combination of restriction patterns with the restriction enzymes used. This study uses and extends this profile naming scheme, but where novel patterns have been discovered the lettering has been continued consecutively and the profiles are named in the format X (Y). X (Y) represents the combination of the individual restriction patterns of *omp *2a and 2b genes with the *omp*2a gene profiles shown in parenthesis.

### IS*711 *fingerprinting

The mobile genetic element IS*711 *has proven a useful target for molecular characterisation based on the number and distribution of IS*711 *copies within the bacterial genomes. A digoxigenin-labelled IS*711 *probe was generated using primers sequences 5' GACCAAGCTGCATGCTG 3' and 5' TGCGAGATGGACGAAGC 3' derived from methods and sequences previously described by Halling *et al*., (1993) [47] and Ouahrani *et al *(1993) [27]. Genomic DNA from each strain was incubated at 37°C for 3 hours with 40 U of *Eco*RI (Promega) before electrophoresis through a 0.8% (w/v) agarose gel at 50 V overnight. The fragments were transferred to Hybond-N nitrocellulose (Amersham Pharmacia Biotech) using a vacuum blot (BioRad) and hybridised overnight at 65°C with the labelled IS*711 *probe. The hybridised probe was detected using alkaline phosphatase labelled anti-digoxigenin antibody (Roche). Reaction with the chemiluminescent substrate (CSPD) (Roche) was measured by exposure to X-ray film (Amersham). RFLP profiles were analysed using Bionumerics (Version 4.5, Applied Maths) using the following tolerance settings: optimisation 0%, and position tolerance 1%. Profiles were clustered using Dice's coefficients and the UPGMA approach.

### IRS-Derivative PCR

Strains in this study were analysed by a derivative of the Infrequent Restriction Site-PCR (IRS-PCR), using a method described by Cloeckaert *et al*., (2003) [48]. Primers designed for PCRs II, III, and IV contain portions of the IS*711 *element and are intended to be specific for cetacean isolates. PCR I is intended to be specific for isolates from seals.

## Authors' contributions

CD carried out the *omp *PCR RFLP and IRS-Derivative PCR studies and drafted the manuscript. ES carried out the IS*711 *fingerprinting studies. LP carried out the classical typing. JS, JB and AW helped draft the manuscript. AM conceived of the study, and participated in its design and coordination and helped to draft the manuscript. AK participated in the *omp *PCR RFLP and IRS-Derivative PCR studies. All authors read and approved the final manuscript.

## Supplementary Material

Additional File 1 **Test results of strains included in the study.** The data provided shows phenotypic and molecular characterisation of marine mammal isolates included in this study. ^1 ^PCR I, specific for isolates from pinnipeds. PCR II, III and IV specific for isolates from cetaceans. ^2 ^Overall endonuclease restriction pattern profiles of *omp *2a gene and 2b gene with *omp *2a gene profiles shown in parenthesis. ^3 ^Berkeley Webridge L = Lysis NL = No. ^4 ^Webridge L = Lysis NL = No. ^5 ^Firenze Webridge L = Lysis NL = No. ^6 ^Tibilisi Webridge L = Lysis NL = No. ^7 ^CO_2 _requirement. ^8 ^Basic fuchsin at 20 μl/ml (1/50,000 w/v). ^9 ^Thionin at 20 μl/ml (1/50,000 w/v). ^10 ^Agglutination with monospecific sera. ^11 ^Location At = Atlantic ocean, Pa = Pacific ocean. Click here for file

## References

[B1] Ross HM, Foster G, Reid RJ, Jahans KL, MacMillan AP (1994). *Brucella *species infection in sea-mammals. Vet Rec.

[B2] Ewalt DR, Payeur JB, Martin BM, Cummins DR, Miller WG (1994). Characteristics of a *Brucella *species from a bottlenose dolphin (*Tursiops truncatus*). J Vet Diagn Invest.

[B3] Foster G, Jahans KL, Reid RJ, Ross HM (1996). Isolation of *Brucella *species from cetaceans, seals and an otter. Vet Rec.

[B4] Jahans KL, Foster G, Broughton ES (1997). The characterisation of *Brucella *strains isolated from marine mammals. Vet Microbiol.

[B5] Garner MM, Lambourn DM, Jeffries SJ, Hall PB, Rhyan JC, Ewalt DR, Polzin LM, Cheville NF (1997). Evidence of *Brucella *infection in Parafilaroides lungworms in a Pacific harbor seal *(Phoca vitulina richardsii*). J Vet Diagn Invest.

[B6] Clavareau C, Wellemans V, Walravens K, Tryland M, Verger JM, Grayon M, Cloeckaert A, Letesson JJ, Godfroid J (1998). Phenotypic and molecular characterization of a *Brucella *strain isolated from a Minke Whale (*Balaenoptera acutorostrata*). Microbiology.

[B7] Foster G, MacMillan AP, Godfroid J, Howie F, Ross HM, Cloeckaert A, Reid RJ, Brew S, Patterson IAP (2002). A review of *Brucella *sp. infection of sea mammals with particular emphasis on isolates from Scotland. Vet Microbiol.

[B8] Ross HM, Jahans KL, MacMillan AP, Reid RJ, Thompson PM, Foster G (1996). *Brucella *species infection in North Sea seal and cetacean populations. Vet Rec.

[B9] Jepson PD, Brew S, MacMillan AP, Baker JR, Barnett J, Kirkwood JK, Kuiken T, Robinson IR, Simpson VR (1997). Antibodies to *Brucella *VR:in marine mammals around the coast of England and Wales. Vet Rec.

[B10] Tryland M, Kleivane L, Alfredson A, Kjeld M, Arnason A, Stuen S, Godfroid J (1999). Evidence of *Brucella *infection in marine mammals in the North Atlantic Ocean. Vet Rec.

[B11] VanBressam MF, van Waerebeek K, Raga JA, Godfroid J, Brew SD, MacMillan AP (2001). Serological evidence of *Brucella *species in odontocetes from the south Pacific and the Mediterranean. Vet Rec.

[B12] Nielsen O, Nielsen K, Stewart REA (1996). Serological evidence of *Brucella *spp. Exposure in Atlantic walruses (*Odobenus rosmarus rosmarus*) and ringed seals (*Phoca hispida*) of Arctic Canada. Arctic.

[B13] Nielsen O, Stewart REA, Nielsen K, Measures L, Duignan P (2001). Serological survey of *Brucella *spp. antibodies in some marine mammals of North America. J Wildlife Dis.

[B14] Ohishi K, Zenitani R, Bando Takeharu, Goto Y, Uchida K, Maruyama T, Yamamoto S, Miyazaki N, Fujise Y (2003). Pathological and Serological evidence of *Brucella*-infection in baleen whales (*Mysticeti*) in the western North Pacific. Comp Immunol Microb.

[B15] Nielsen O, Nielsen K, Braun R, Kelly L (2005). A comparison of four serologic assays in screening for *Brucella *exposure in Hawaiian monk seals. J Wildl Dis.

[B16] Tachibana M, Watanabe K, Kim S, Omata Y, Murata K, Hammond T, Watarai M (2006). Antibodies to *Brucella *spp. in Pacific Bottlenose Dolphins from the Solomon Islands. J Wildl Dis.

[B17] Retamal P, Blank O, Abalos P, Torres D (2000). Detection of anti-*Brucella *antibodies in pinnipeds from the Antarctic territory. Vet Rec.

[B18] Dawson CE (2005). Anti-*Brucella *antibodies in pinnipeds of Australia. Microbiology Aust.

[B19] Alton GG, Jones LM, Angus RD, Verger JM (1988). Techniques for the Brucellosis Laboratory. Institut National de la Recherche Agronomique, Paris.

[B20] Corbel MJ, Brinley-Morgan WJ, Krieg NR, Holt JG (1984). Genus *Brucella *Meyer and Shaw 1920. 173^AL^. Bergey's Manual of Systematic Bacteriology.

[B21] Hoyer BH, McCullough NB (1968). Polynucleotide homologues of *Brucella *deoxyribonucleic acids. J Bacteriol.

[B22] Hoyer BH, McCullough NB (1968). Homologues of deoxyribonucleic acids from *Brucella ovis*, canine abortion organisms and other *Brucella *species. J Bacteriol.

[B23] Verger JM, Grimont F, Grimont PAD, Grayon M (1985). *Brucella *a monospecific genus as shown by deoxyribonucleic acid hybridisation. Int J Syst Bacteriol.

[B24] Verger JM, Grimont F, Grimont PAD, Grayon M (1987). Taxonomy of the genus *Brucella*. Ann Ins Pasteur Microbiol.

[B25] De Ley J, Mannheim W, Seyers P, Lievens A, Densin M, Vanhoucke M, Gillis M (1987). Ribosomal ribonucleic acid astron similarities and taxonomic neighbourhood of *Brucella *and CDC Group. Int J Syst Bacteriol.

[B26] Bricker J, Halling SM (1994). Differentiation of *Brucella abortus *bv. 1, 2, and 4, *Brucella melitentis*, *Brucella ovis*, and *Brucella suis *bv. 1 by PCR. J Clin Microbiol.

[B27] Ouahrani S, Michaux S, Widada JS, Bourg G, Tournebize R, Ramuz M, Liautard JP (1993). Identification and sequence analysis of IS*6501*, an insertion sequence in *Brucella *spp.: relationship between genomic structure and number of IS*6501 *copies. J Gen Microbiol.

[B28] Cloeckaert A, Verger JM, Grayon M, Grepinet O (1995). Restriction site polymorphism of the genes encoding the major 25 kDa and 36 kDa outer membrane proteins of *Brucella*. Microbiology.

[B29] Whatmore AM, Murphy TJ, Shankster S, Young E, Cutler SJ, MacMillan AP (2005). Use of Amplified Fragment Length Polymorphism to identify and type *Brucella *isolates of medical and veterinary interest. J Clin Microbiol.

[B30] García-Yoldi D, Marín CM, de Miguel MJ, Muñoz PM, Vizmanos JL, López-Goñi I (2006). Multiplex PCR assay for the identification and differentiation of all *Brucella *species and the vaccine strains *Brucella abortus *S19 and RB51 and *Brucella melitensis *REV1. Clin Chem.

[B31] Ratushna V, Sturgill D, Ramamoorthy S, Reichow S, He Y, Lathigra R, Sriranganathan N, Halling S, Boyle S, Gibas C (2006). Molecular targets for rapid identification of *Brucella *spp. BMC Microbiol.

[B32] Whatmore M, Perrett L, MacMillan AP (2007). Characterisation of the genetic diversity of *Brucella *by multilocus sequencing. BMC Microbiol.

[B33] Le Flèche P, Jaques I, Grayon M, Al Dahouk S, Bouchon P, Denoeud F, Nöckler K, Neubauer H, Guilloteau LA, Vergnaud G (2006). Evaluation and selection of tandem repeat loci for a *Brucella *MLVA typing assay. BMC Microbiol.

[B34] Whatmore AM, Shankster SJ, Perrett LL, Murphy TJ, Brew SD, Thirlwall RE, Cutler SJ, MacMillan AP (2006). Identification and characterisation of Variable-Number Tandem-Repeat markers for typing of *Brucella *spp. J Clin Microbiol.

[B35] Bricker BJ, Ewalt DR, MacMillan AP, Foster G, Brew S (2000). Molecular characterization of *Brucella *strains isolated from marine mammals. J Clin Microbiol.

[B36] Foster G, Osterman BS, Godfroid J, Jacques I, Cloeckaert A (2007). *Brucella *ceti sp. nov. and *Brucella *pinnipedialis sp. nov. for *Brucella *strains with cetaceans and seals as their preferred hosts. Int J Syst Evol Microbiol.

[B37] Miller WG, Adams LG, Ficht TA, Cheville NF, Payeur JP, Harley DR, House C, Ridgeway SH (1999). *Brucella *– induced abortions and infections in bottlenose dolphins (*Tursiops truncatus*). J Zoo Wildlife Med.

[B38] Jensen AE, Cheville NF, Thoen CO, MacMillan AP, Miller WG (1999). Genomic fingerprinting and development of a dendrogram for *Brucella *spp. isolated from seals, porpoises and dolphins. J Vet Diagn Invest.

[B39] Groussaud P, Shankster S, Koylass MS, Whatmore AM (2007). Molecular typing divides marine mammal strains of *Brucella *into at least three groups with distinct host preferences. J Med Microbiol.

[B40] Bourg G, O'Callaghan D, Boschiroli ML (2007). The genomic structure of *Brucella *strains isolated from marine mammals gives clues to evolutionary history within the genus. Vet Microbiol.

[B41] McDonald WL, Jamaludin R, Macereth G, Hansen M, Humphrey S, Short P, Taylor T, Swingler J, Dawson CE, Whatmore AM, Stubberfield E, Perrett LL, Simmons G (2006). Characterisation of a *Brucella *sp. strain as a marine-mammal type despite isolation from a patient with spinal osteomyelitis in New Zealand. J Clin Microbiol.

[B42] Sohn A, Probert A, Glaser C, Gupta N, Bollen A, Wong J, Grace E, McDonald W (2003). Human neorobrucellosis with intracerebral granuloma caused by a marine mammal *Brucella *spp. Emerg Infect Dis.

[B43] Whatmore AM, Dawson C, Groussaud P, Koylass M, King A, Shankster S, Sohn A, Probert W, McDonald W (2008). A marine mammal *Brucella *genotype associated with zoonotic infection. Emerg Inf Dis.

[B44] Ohishi K, Takishita K, Kawato M, Zenitani R, Bando T, Fujise Y, Goto Y, Yamamoto S, Maruyama T (2004). Molecular evidence of new variant *Brucella *in North Pacific common minke whales. Microbe Infect.

[B45] Hernández-Mora G, González-Barrientos R, Morales J-A, Chaves-Olarte E, Guzmán-Verri C, Baquero-Calvo E, De-Miguel M-J, Marin C-M, Blasco J-M, Moreno E (2008). Neorobrucellosis in stranded dolphins, Costa Rica. Emerg Infect Dis.

[B46] Cloeckaert A, Verger J-M, Grayon M, Paquet J-Y, Garin-Bastiji B, Foster G, Godfroid J (2001). Classification of *Brucella *spp. isolated from marine mammals by DNA polymorphism at the *omp*2 locus. Microbe Infect.

[B47] Halling SM, Tatum FM, Bricker BJ (1993). Sequence and characterisation of an insertion sequence, IS*711*, from *Brucella ovis*. Gene.

[B48] Cloeckaert A, Grayon M, Grepinet O, Bourmedine KS (2003). Classification of *Brucella *strains isolated from marine mammals by infrequent restriction site-PCR and development of specific PCR identification tests. Microbe Infect.

