# Supplementary material for: Phenotypic and molecular characterisation of Brucella isolates from marine mammals
Source: BMC Microbiol. 2008 Dec 17;8:224. doi: 10.1186/1471-2180-8-224 (PMC2647937; doi:10.1186/1471-2180-8-224)
Supplement: Additional File 1 — Test results of strains included in the study. The data provided shows phenotypic and molecular characterisation of marine mammal isolates included in this study. 1 PCR I, specific for isolates from pinnipeds. PCR II, III and IV specific for isolates from cetaceans. 2 Overall endonuclease restriction pattern profiles of omp 2a gene and 2b gene with omp 2a gene profiles shown in parenthesis. 3 Berkeley Webridge L = Lysis NL = No. 4 Webridge L = Lysis NL = No. 5 Firenze Webridge L = Lysis NL = No. 6 Tibilisi Webridge L = Lysis NL = No. 7 CO2 requirement. 8 Basic fuchsin at 20 μl/ml (1/50,000 w/v). 9 Thionin at 20 μl/ml (1/50,000 w/v). 10 Agglutination with monospecific sera. 11 Location At = Atlantic ocean, Pa = Pacific ocean. [file 1471-2180-8-224-S1.doc]

| **Sample Ref** | | **Host** | **Location** | **IRS PCR^1^** | | | | **IS*7II* Fingerprinting** | **OMP Pattern^2^** | Phage Lysis | | | | **Growth Characteristics** | | | | |
| --- | --- | --- | --- | --- | --- | --- | --- | --- | --- | --- | --- | --- | --- | --- | --- | --- | --- | --- |
|  |  |  |  | **I** | **II** | **III** | **IV** |  |  | **BK_2_^3^** | **WB^4^** | **FI^5^** | **TB^6^** | **CO_2_^7^** | **BF^8^** | **Th^9^** | **A^10^** | **M^10^** |
| **1** | M39/94 | Harbour porpoise | UK | - | + | + | - | Cluster 2 | M(J) | L | L | L | NL | - | + | + | + | + |
| **2** | 52/94 | Harbour porpoise | UK | - | + | + | - | Cluster 2 | M(J) | L | L | L | NL | - | + | + | + | - |
| **3** | M1661/94 | Harbour porpoise | UK | - | + | + | - | Cluster 2 | M(J) | L | L | L | NL | - | + | + | + | - |
| **4** | M51/96 | Harbour porpoise | UK | - | + | + | - | Cluster 2 | M(J) | L | L | L | NL | - | + | + | + | - |
| **5** | M563/99/2 | Harbour porpoise | UK | - | + | + | - | Cluster 2 | M(J) | L | L | L | NL | - | + | + | + | - |
| **6** | 219/00 | Harbour porpoise | UK | - | + | + | - | Cluster 2 | M(J) | L | L | L | NL | + | + | + | + | - |
| **7** | M187/00 | Atlantic white-sided dolphin | UK | - | + | + | - | Cluster 2 | M(J) | L | L | L | NL | - | - | - | + | - |
| **8** | 096/01 | Harbour porpoise | UK | - | + | + | - | Cluster 2 | M(J) | L | L | L | NL | - | + | + | + | - |
| **9** | M46/01 | Harbour porpoise | UK | - | + | + | - | Cluster 2 | M(J) | L | L | L | NL | - | - | + | + | - |
| **10** | M70/02/1 | Harbour porpoise | UK | - | + | + | - | Cluster 2 | M(J) | L | L | L | NL | - | - | - | + | - |
| **11** | B202R | Minke whale | Norway | - | + | + | - | Cluster 2 | M(J) | L | L | L | NL | - | + | + | + | + |
| **12** | M130/02/1 | Harbour porpoise | UK | - | + | + | - | Cluster 2 | M(J) | L | L | L | NL | - | + | + | + | + |
| **13** | M490/95 | Common seal | UK | - | + | + | - | Cluster 2 | M(J) | L | L | NL | NL | - | + | + | + | - |
| **14** | F23/97 | Dolphin sp. | France | - | + | + | - | Cluster 2 | M(J) | L | L | NL | NL | - | + | + | + | - |
| **15** | M39/1/04 | Harbour porpoise | UK | - | + | + | - | Cluster 2 | M(J) | L | L | NL | NL | - | - | - | + | - |
| **16** | M23/03/4 | Harbour porpoise | UK | - | + | + | - | Cluster 2 | M(J) | L | L | NL | NL | - | + | + | + | - |
| **17** | M89/03/5 | Harbour porpoise | UK | - | + | + | - | Cluster 2 | M(J) | L | L | NL | NL | - | + | + | + | - |
| **18** | M51/04/2 | Harbour porpoise | UK | - | + | + | - | Cluster 2 | M(J) | L | L | NL | NL | - | + | + | + | - |
| **19** | M93/04/3 | Harbour porpoise | UK | - | + | + | - | Cluster 2 | M(J) | L | L | NL | NL | - | + | + | + | - |
| **20** | M100/04/6 | Harbour porpoise | UK | - | + | + | - | Cluster 2 | M(J) | L | L | NL | NL | + | - | + | + | - |
| **21** | M117/01/1 | Harbour porpoise | UK | - | + | + | - | Cluster 2 | M(J) | L | NL | L | NL | - | + | + | + | - |
| **22** | M217/03 | Harbour porpoise | UK | - | + | + | - | Cluster 2 | M(J) | L | NL | L | NL | - | + | + | + | - |
| **23** | M194/10/04 | Harbour porpoise | UK | - | + | + | - | Cluster 2 | M(J) | L | NL | L | NL | - | + | + | + | - |
| **24** | M452/97/2 | Common dolphin | UK | - | + | + | - | Cluster 2 | M(J) | L | NL | NL | NL | - | + | + | + | - |
| **25** | M499/99/4 | Harbour porpoise | UK | - | + | + | - | Cluster 2 | M(J) | L | NL | NL | NL | - | + | + | + | - |
| **26** | M150/03/3 | Harbour porpoise | UK | - | + | + | - | Cluster 2 | M(J) | L | NL | NL | NL | - | + | + | + | - |
| **27** | M165/03/6 | Harbour porpoise | UK | - | + | + | - | Cluster 2 | M(J) | L | NL | NL | NL | - | + | + | + | - |
| **28** | M291/03/2 | Harbour porpoise | UK | - | + | + | - | Cluster 2 | M(J) | L | NL | NL | NL | - | + | + | + | - |
| **29** | M2/00 | Atlantic white-sided dolphin | UK | - | + | + | - | Cluster 2 | M(J) | NL | NL | L | NL | - | + | + | + | - |
| **30** | M854/98 | Harbour porpoise | UK | - | + | + | - | Cluster 2 | M(J) | L | L | L | L | - | + | + | + | - |
| **31** | M575/99/4 | Harbour porpoise | UK | - | + | + | - | Cluster 2 | M(J) | L | L | L | NL | - | + | + | + | - |
| **32** | M615/99 | Harbour porpoise | UK | - | + | + | - | Cluster 2 | M(J) | L | L | L | NL | - | + | + | + | - |
| **33** | M610/99 | Harbour porpoise | UK | - | + | + | - | Cluster 2 | M(J) | L | NL | L | L | - | + | + | + | - |
| **34** | M49/01 | Harbour porpoise | UK | - | + | + | - | Cluster 2 | M(J) | NL | NL | NL | L | - | + | + | + | - |
| **35** | 026/96 | Harbour porpoise | UK | - | + | + | - | Cluster 2 | M(J) | NL | NL | NL | NL | - | + | + | + | - |
| **36** | 438/97 | Harbour porpoise | UK | - | + | + | - | Cluster 2 | M(J) | NL | NL | NL | NL | - | + | + | + | - |
| **37** | M493/99/1 | Harbour Porpoise | UK | - | + | + | - | Cluster 2 | M(J) | NL | NL | NL | NL | - | + | + | + | - |
| **38** | M52/01 | Atlantic white-sided dolphin | UK | - | + | + | - | Cluster 2 | M(J) | NL | NL | NL | NL | - | + | + | + | - |
| **39** | M195/03/10 | Harbour porpoise | UK | - | + | + | - | Cluster 2 | M(J) | NL | NL | NL | NL | - | + | + | + | - |
| **40** | PP960213-3 | Harbour porpoise | Germany | - | + | + | - | Cluster 2 | M(J) | L | NL | L | NL | - | + | + | + | - |
| **41** | M14/8/98 | Harbour porpoise | UK | - | + | + | - | Cluster 2 | M(J) | L | L | L | NL | - | + | + | + | - |
| **42** | M466/3/04 | Common seal | UK | - | + | + | - | Cluster 2 | M(J) | L | L | L | NL | - | + | + | + | - |
| **43** | M997/94 | Atlantic white-sided dolphin | UK | - | + | + | - | Cluster 2 | M(J) | L | L | NL | NL | - | + | + | + | - |
| **44** | M2788/97 | Atlantic white-sided dolphin | UK | - | + | + | - | Cluster 2 | M(J) | L | L | NL | NL | - | + | + | + | - |
| **45** | M103/99 | Harbour porpoise | UK | - | + | + | - | Cluster 2 | M(J) | L | L | NL | NL | - | + | + | + | - |
| **46** | M21/4/04 | Harbour porpoise | UK | - | + | + | - | Cluster 2 | M(J) | L | L | NL | NL | - | + | + | + | - |
| **47** | M38/04/3 | Harbour porpoise | UK | - | + | + | - | Cluster 2 | M(J) | L | L | NL | NL | - | + | + | + | - |
| **48** | M1068/91 | Harbour porpoise | UK | - | + | + | - | Cluster 2 | M(J) | L | NL | L | NL | - | + | + | + | + |
| **49** | M12/00/3 | Harbour porpoise | UK | - | + | + | - | Cluster 2 | M(J) | L | L | L | L | - | + | + | + | - |
| **Sample Ref** | | **Host** | **Location** | **IRS PCR^1^** | | | | **IS*7II* Fingerprinting** | **OMP Pattern^2^** | Phage Lysis | | | | **Growth Characteristics** | | | | |
|  |  |  |  | **I** | **II** | **III** | **IV** |  |  | **BK_2_^3^** | **WB^4^** | **FI^5^** | **TB^6^** | **CO_2_^7^** | **BF^8^** | **Th^9^** | **A^10^** | **M^10^** |
| **50** | M1747/98/3 | Harbour Porpoise | UK | - | + | + | - | Cluster 2 | M(J) | L | NL | L | L | - | + | + | + | - |
| **51** | M2328/4/97 | White-beaked dolphin | UK | - | + | + | - | Cluster 2 | M(J) | NL | NL | NL | NL | - | + | + | + | - |
| **52** | M12/04/5 | Harbour porpoise | UK | - | + | + | - | Cluster 2 | M(J) | L | L | L | NL | - | + | + | + | - |
| **53** | M311/03/05 | Harbour porpoise | UK | - | + | + | - | Cluster 2 | M(J) | L | NL | NL | NL | - | + | + | + | - |
| **54** | M624/99/2 | Striped dolphin | UK | - | - | - | + | Cluster 3 | N(K) | L | L | L | NL | + | + | + | + | - |
| **55** | M656/99 | Striped dolphin | UK | - | - | - | + | Cluster 3 | N(K) | L | L | L | NL | - | + | + | + | - |
| **56** | M9/02/1 | Striped dolphin | UK | - | - | - | + | Cluster 3 | N(K) | L | L | L | NL | - | + | + | + | - |
| **57** | M260/03/1 | Atlantic white-sided dolphin | UK | - | - | - | + | Cluster 3 | N(K) | L | L | L | NL | - | + | + | + | - |
| **58** | M22/02/1 | Striped dolphin | UK | - | - | - | + | Cluster 3 | N(K) | L | L | NL | NL | + | + | + | + | + |
| **59** | M2194/94 | Striped dolphin | UK | - | - | - | + | Cluster 3 | N(K) | L | L | L | L | - | + | + | + | + |
| **60** | F5/06 | Striped dolphin | Spain | - | - | - | + | Cluster 3 | N(K) | L | L | L | NL | - | + | + | + | - |
| **61** | M40/95 | Striped dolphin | UK | - | - | - | + | Cluster 3 | N(K) | L | L | L | NL | - | + | + | + | - |
| **62** | M49/1/05 | Common dolphin | UK | - | - | - | + | Cluster 3 | N(K) | L | L | L | L- | + | + | + | + | + |
| **63** | M151/12/04 | Bottlenose dolphin | UK | - | - | - | + | Cluster 3 | N(K) | L | L | L | NL | - | + | + | + | - |
| **64** | M66/3/02 | Common dolphin | UK | - | - | - | + | Cluster 3 | N(K) | L | NL | NL | NL | - | + | + | + | + |
| **65** | M654/99/1 | Common dolphin | UK | - | - | - | + | Cluster 3 | N(K) | L | L | L | NL | - | + | + | + | + |
| **66** | M644/93 | Striped dolphin | UK | - | - | - | + | Cluster 3 | N(K) | L | L | L | NL | - | + | + | + | - |
| **67** | M181/97/2 | Atlantic white-sided dolphin | UK | - | - | - | + | Cluster 3 | N(K) | L | L | L | NL | - | + | + | + | - |
| **68** | M194/00/1 | Grey seal | UK | + | - | - | - | Cluster 1 | L(I) | L | L | L | NL | + | + | + | + | - |
| **69** | M374/02/5 | Common seal | UK | + | - | - | - | Cluster 1 | L(I) | L | L | NL | NL | - | + | + | + | - |
| **70** | M2357/93 | Common seal | UK | + | - | - | - | Cluster 1 | L(I) | L | NL | L | NL | + | + | + | + | - |
| **71** | M599/02/1 | Common seal | UK | + | - | - | - | Cluster 1 | L(I) | L | L | L | NL | + | + | + | + | + |
| **72** | M2466/93 | Common seal | UK | + | - | - | - | Cluster 1 | L(I) | L | L | NL | NL | + | + | + | + | - |
| **73** | M1771/94 | European otter | UK | + | - | - | - | Cluster 1 | L(I) | L | L | NL | NL | + | + | + | + | - |
| **74** | M2375/94 | Grey seal | UK | + | - | - | - | Cluster 1 | L(I) | L | L | L | NL | + | + | + | + | - |
| **75** | M514/9677 | Common seal | UK | + | - | - | - | Cluster 1 | L(I) | L | L | NL | NL | + | + | + | + | - |
| **76** | M445/99/2 | Common seal | UK | + | - | - | - | Cluster 1 | L(I) | L | L | NL | NL | + | + | + | - | - |
| **77** | C/02/16559 | Seal sp. | N Ireland | + | - | - | - | Cluster 1 | L(I) | L | L | NL | NL | + | + | + | + | - |
| **78** | C/02/14397 | Seal sp. | N Ireland | + | - | - | - | Cluster 1 | L(I) | L | L | NL | NL | + | + | + | + | - |
| **79** | C/02/15814 | Seal sp. | N.Ireland | + | - | - | - | Cluster 1 | L(I) | L | L | NL | NL | + | + | + | + | - |
| **80** | M336/94 | Common seal | UK | + | - | - | - | Cluster 1 | L(I) | L | NL | NL | NL | + | + | + | + | + |
| **81** | M339/94 | Common seal | UK | + | - | - | - | Cluster 1 | L(I) | L | NL | NL | NL | + | + | + | + | - |
| **82** | M972/94 | Common seal | UK | + | - | - | - | Cluster 1 | L(I) | L | NL | NL | NL | + | + | + | + | - |
| **83** | M13/01/1 | Common seal | UK | + | - | - | - | Cluster 1 | L(I) | L | NL | NL | NL | - | + | + | + | - |
| **84** | C/02/13840 | Seal sp. | N.Ireland | + | - | - | - | Cluster 1 | L(I) | L | NL | NL | NL | + | + | + | + | - |
| **85** | M621/99 | Grey seal | UK | + | - | - | - | Cluster 1 | L(I) | L | NL | NL | L | + | + | + | + | + |
| **86** | C/02/15549 | Seal sp. | N.Ireland | + | - | - | - | Cluster 1 | L(I) | L | L | NL | NL | + | + | + | + | - |
| **87** | M192/00/1 | Minke whale | UK | + | - | - | - | Cluster 1 | O(I) | L | L | L | NL | + | + | + | + | - |
| **88** | M2533/93 | Common seal | UK | + | - | - | - | Cluster 1 | O(I) | L | L | NL | NL | + | + | + | + | - |
| **89** | M342/02/2 | Common seal | UK | + | - | - | - | Cluster 1 | O(I) | L | L | NL | NL | - | + | + | + | - |
| **90** | M449/02/2 | Common seal | UK | + | - | - | - | Cluster 1 | O(I) | L | L | NL | NL | + | + | + | + | - |
| **91** | M250/02/3 | Common seal | UK | + | - | - | - | Cluster 1 | O(I) | L | L | NL | NL | + | + | + | + | - |
| **92** | M305/02/1 | Common seal | UK | + | - | - | - | Cluster 1 | O(I) | L | L | NL | NL | + | + | + | + | - |
| **93** | M292/94 | Common seal | UK | + | - | - | - | Cluster 1 | O(I) | L | NL | L | NL | + | + | + | + | - |
| **94** | M163/99 | Hooded seal | UK | + | - | - | - | Cluster 4 | P (I) | L | L | L | L | - | + | + | + | - |
| **95** | M2006/94 | Hooded seal | UK | + | - | - | - | Cluster 4 | P (I) | NL | L | L | L | + | + | + | + | - |
| **96** | M603/99 | Hooded seal | UK | - | - | - | - | Cluster 4 | P (I) | L | L | L | L | + | + | + | + | - |
| **Sample Ref** | | **Host** | **Location** | **IRS PCR^1^** | | | | **IS*7II* Fingerprinting** | **OMP Pattern^2^** | Phage Lysis | | | | **Growth Characteristics** | | | | |
|  |  |  |  | **I** | **II** | **III** | **IV** |  |  | **BK_2_^3^** | **WB^4^** | **FI^5^** | **TB^6^** | **CO_2_^7^** | **BF^8^** | **Th^9^** | **A^10^** | **M^10^** |
| **97** | 2-1350 | Bottlenose dolphin | USA Pa^11^ | + | - | - | - | Cluster 1 | Q(I) | L | NL | NL | NL | - | + | + | - | - |
| **98** | 97-269 | Common seal | USA At | + | - | - | - | Cluster 1 | O(I) | L | NL | NL | NL | - | + | + | - | - |
| **99** | 98-230 | Bottlenose dolphin | USA Pa | + | - | - | - | Cluster 1 | O(I) | L | NL | NL | NL | - | + | + | - | - |
| **100** | 96-408 | Common seal | USA At | - | - | - | - | Cluster 1 | O(I) | L | NL | NL | NL | - | + | + | - | - |
| **101** | 96-566 | Common seal | USA At | - | - | - | - | Cluster 1 | O(I) | L | NL | NL | NL | - | + | + | - | - |
| **102** | 97-324 | Common seal | USA At | - | - | - | - | Cluster 1 | O(I) | NL | NL | NL | NL | - | + | + | - | - |
